# Disturbances in Nitric Oxide Cycle and Related Molecular Pathways in Clear Cell Renal Cell Carcinoma

**DOI:** 10.3390/cancers15245797

**Published:** 2023-12-11

**Authors:** Corina Daniela Ene, Mircea Tampa, Simona Roxana Georgescu, Clara Matei, Iulia Maria Teodora Leulescu, Claudia Ioana Dogaru, Mircea Nicolae Penescu, Ilinca Nicolae

**Affiliations:** 1 Department of Nephrology, Carol Davila Clinical Hospital of Nephrology, 010731 Bucharest, Romania; koranik85@yahoo.com (C.D.E.); mirceapenescu4@gmail.com (M.N.P.); 2 Department of Nephrology, “Carol Davila” University of Medicine and Pharmacy, 020021 Bucharest, Romania; 3 Department of Dermatology, “Carol Davila” University of Medicine and Pharmacy, 020021 Bucharest, Romania; matei_clara@yahoo.com; 4 Department of Dermatology, “Victor Babes” Clinical Hospital for Infectious Diseases, 030303 Bucharest, Romania; iulialeulescu@gmail.com (I.M.T.L.); claudia_ioana97@yahoo.com (C.I.D.); drnicolaei@yahoo.ro (I.N.)

**Keywords:** nitric oxide, signalling, ccRCC

## Abstract

**Simple Summary:**

In this article, we analyze the current state of research on nitric oxide biosynthesis metabolites in clear cell renal cell carcinoma and the metabolic pathways that amplify nitric oxide production in the tumour microenvironment. Special attention is given to nitric oxide homeostasis disruption, mechanisms of nitric oxide biosynthesis and signalling, analysis of nitric oxide bimodal effects, quantitative analysis of nitric oxide, metabolites ureagenic cycle and glutamine metabolism, arginine metabolism and depletion, hyperammonemia, branched-chain amino acids catabolism and nitric oxide-based therapy for cancer. Clarifying these issues will contribute to the development of personalized medicine for patients with clear cell renal carcinoma.

**Abstract:**

It is important to note that maintaining adequate levels of nitric oxide (NO), the turnover, and the oxidation level of nitrogen are essential for the optimal progression of cellular processes, and alterations in the NO cycle indicate a crucial step in the onset and progression of multiple diseases. Cellular accumulation of NO and reactive nitrogen species in many types of tumour cells is expressed by an increased susceptibility to oxidative stress in the tumour microenvironment. Clear cell renal cell carcinoma (ccRCC) is a progressive metabolic disease in which tumour cells can adapt to metabolic reprogramming to enhance NO production in the tumour space. Understanding the factors governing NO biosynthesis metabolites in ccRCC represents a relevant, valuable approach to studying NO-based anticancer therapy. Exploring the molecular processes mediated by NO, related disturbances in molecular pathways, and NO-mediated signalling pathways in ccRCC could have significant therapeutic implications in managing and treating this condition.

## 1. Introduction

It has recently been noted that the evolution of renal tumours is regulated at the molecular level, and the impact of metabolic remodelling on tumour biology is largely unknown [1,2,3,4].

Renal cell carcinoma (RCC) affects 400,000 patients annually worldwide, causing 2.4/100,000 deaths [5]. Clear cell renal carcinoma (ccRCC) is the most common type of kidney cancer, accounting for 70–75% of RCC cases [5,6]. ccRCC originates from tubular epithelial cells and is orchestrated by epigenetic alterations. Genomic loss of the Von Hippel-Lindau (VHL) tumour suppressor, arginase 2, argininosuccinate synthetase (enzymes involved in the urea cycle), the progression of arginine, glutamine, tryptophan, glutathione, cysteine/methionine metabolism, polyamine, and activation of HIF2a-mediated pro-oncogenic signalling are the most well-known metabolic disturbances in ccRCC [1,3,7].

ccRCC is a progressive metabolic disease in which tumour cells adapt to interconnected metabolic and epigenetic reprogramming, along with alterations in nitrogen homeostasis [5,8,9,10]. Tumours actively modulate the host’s metabolism to increase their nitrogen supply and promote their growth and progression [4,10,11,12,13].

The regulation of nitrogen supply in renal tumours is a complex process and is influenced by disruptions in NO homeostasis, ureagenic cycle dysregulation, low levels of arginine, loss of VHL, mitochondrial dysfunction, HIF expression, disruption of branched-chain amino acid metabolism, nucleotide synthesis, the presence of endogenous competitive NOS inhibitors [1,3,4,5,6,12,13,14].

In this article, we analyze the current state of research on NO biosynthesis metabolites in ccRCC and the metabolic pathways that amplify NO production in the tumour microenvironment. Understanding the role of NO biosynthesis in the pathogenesis of ccRCC helps in identifying effective therapeutic means for the prevention and treatment of diseases associated with the alteration of the L-arginine–NO molecular pathway.

## 2. The Disruption of NO Homeostasis in ccRCC

NO has emerged as a molecule of interest in carcinogenesis and tumour progression due to its bimodal role in various cellular processes [4]. Tumour cells require distinct NO concentrations, promoting either a pro-tumoural phenotype or an anti-tumoural phenotype. Low to moderate levels may promote tumourigenesis, whilst higher levels would exert anti-tumour effects [8]. The effects of NO in cancer cells appear to depend on the origin of NO, the type, activity, and localization of NOS isoforms, concentration, and duration of NO exposure, temporal and spatial regulation at various levels of the NO cycle, NO-mediated signalling pathways, the metabolic phenotype of cells in the tumour microenvironment, and cellular sensitivity to NO. In the tumour microenvironment, low to moderate levels of NO (NO) derived from tumour and endothelial cells can activate angiogenesis, promoting an aggressive phenotype. Conversely, high levels of NO derived from M1 macrophages and Th1 lymphocytes can exert an anti-tumoural effect, providing protection against cancer [1,8,15,16]. Therefore, identifying the NO equilibrium state is particularly important for ccRCC biology.

### 2.1. The Disruption of NO Biosynthesis in ccRCC

**Figure 1:**
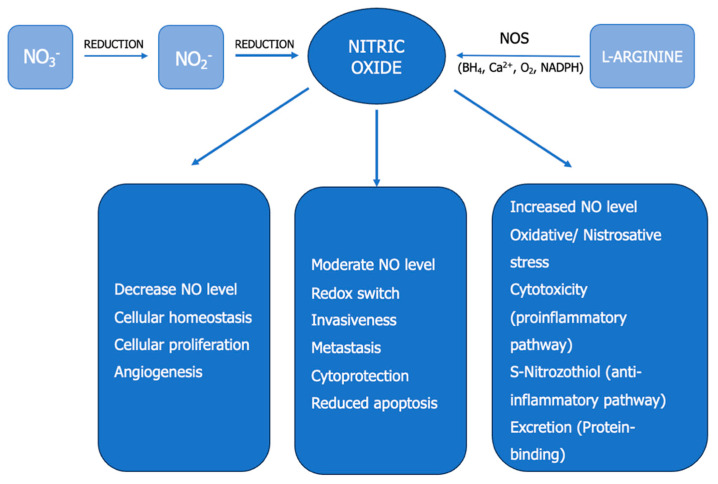
NO biochemistry. Concentration-dependent effects of NO in cancer. NO—nitric oxide, NOS—NO synthase, NO_3_^−^—nitrate, NO_2_^−^—nitrite, NADPH—nicotinamide adenine dinucleotide phosphate, BH4—6R-5,6,7,8-tetrahydrobiopterin.

Currently, there are two major pathways for NO generation in vivo: the L-arginine–NO pathway and the Nitrate–Nitrite–NO pathway (Figure 1) [2,4,17,18].

In the oxidative pathway, NOSs convert plasma L-arginine into NO in equimolar amounts. L-arginine comes from endogenous sources (de novo synthesis, protein turnover) and exogenous sources (diet). Microorganisms carry out the reductive nitrate–nitrite–NO processes at the oral and intestinal levels. Nitrates come from exogenous or endogenous sources (diet, water, environment). Under normoxia, the total daily production of NO in the entire body was estimated at 1100 micromoles. The relative contribution of the classical L-arginine–NO pathway represents 90% of the total NO. In comparison, the average rate of NO synthesis through the alternative nitrate–nitrite–NO pathway is approximately 10% [4,17]. Recent approaches in nephrology attempt to analyze ccRCC and autosomal dominant polycystic kidney disease (ADPKD) as arginine-auxotrophic metabolic diseases. These conditions are associated with impaired capacity for recycling or synthesizing intracellular arginine through the urea cycle pathway [19,20,21]. In malignancy, L-arginine functions as both an onconutrient and an immunonutrient [22]. A recent study has documented the prospective relationship between initial serum arginine concentrations and the risk of cancer in hypertensive participants. Higher serum levels of arginine demonstrate a significantly increased risk of cancer [23].

In ccRCC, NO biosynthesis and the mechanisms that regulate the NO cycle are disrupted. At the renal level, NO levels are regulated through several mechanisms. These include the availability of L-arginine (affecting renal L-arginine biosynthesis, endothelial transport), the competition between NOS and other metabolic pathways for NO utilization, elevated circulating levels of ADMA (asymmetric dimethylarginine), an endogenous competitive NOS inhibitor (increased protein methylation, protein catabolism rate to supply free ADMA, reduced ADMA catabolism via DDAH-dimethylarginine dimethylaminohydrolase), and the distribution of NOS at the renal level [24,25].

Three isoforms of NO synthetases (NOSs) are known to be involved in NO synthesis: endothelial (NOS1 or eNOS), inducible (NOS2 or iNOS), and neuronal (NOS3 or nNOS) [16,26].

A variant of NOS in red blood cells (eNOS-RBC) has recently been studied for its activity in ischemia-reperfusion injury [15,16].

NOSs require nicotinamide adenine dinucleotide phosphate (NADPH), flavin mononucleotide (FMN), flavin adenine dinucleotide (FAD), and 6R-5,6,7,8-tetrahydrobiopterin (BH4) as cofactors. In the kidney, eNOS was identified in vascular endothelium and certain epithelia (thick ascending loop of Henle, collecting duct). At the same time, nNOS and nNOS mRNA were detected in the macula densa, efferent arterioles, collecting duct, Bowman’s capsule, and iNOS was observed in the inner medullary collecting duct, immune cells, and tumour cells [27].

### 2.2. The Disruption of NO Signaling in ccRCC

The molecular mechanisms governing the ubiquitous nature of NO transduction and its impact on biological processes involve, on the one hand, the direct interaction between NO and potential cellular targets and, on the other hand, the formation of other reactive nitrogen species that possess signalling properties themselves [18].

The processes mediated by NO in the tumour environment involve either cyclic guanosine monophosphate (cGMP)-dependent or cGMP-independent reactions. In the cGMP-dependent pathway, NO binds to the heme component of soluble guanylate cyclase (sGC), promoting the conversion of guanosine triphosphate (GTP) to cGMP, activating protein kinases and phosphodiesterases and cyclic nucleotide channels. In the NO/sGC/cGMP pathway, low NO levels produced by constitutive NOSs regulate renal physiology. The phenomenon concludes rapidly after removing the initial stimulus from the tumour microenvironment, explained by the short half-life of NO, estimated at 0.1–2 s [12,15,28]. Disruption of NO/sGC/cGMP signalling results in reduced cGMP levels and the emergence of severe kidney pathologies [29].

In the cGMP-independent pathway, high levels of NO produced by iNOS induce oxidative and nitrosative stress, damage macromolecules, generate mutagenic and carcinogenic compounds (S-nitrosothiols, N-nitrosamines), halt the cell cycle, trigger antiapoptotic responses, senescence, and promote tumour progression. Recent quantified post-translational modifications in ccRCC (glycosylation, TDH, protein carbonylation, proteolytic cleavage, peroxidation, methylation, phosphorylation) accompany tumour development and metastasis promotion [3]. The disruption of the NO cycle in tumours is associated with the alteration of NF-kB, AP-1, and CREB pathways, as well as MAPK/PI3K, Ras, G-proteins, mitochondrial cytochrome c oxidase [12,15,28]. NO acts as a pleiotropic messenger, thus influencing the ccRCC progression.

Among the numerous NO-mediated signalling mechanisms, the interaction of NOSs–NO, the coupling of NOSs-O_2_(−), post-translational protein changes, and epigenetic regulation play a significant role in the ccRCC progression.

The NOSs-NO signalling, which has recently been revealed, affects NO bioavailability through cGMP-dependent mechanisms. Constitutive NOSs modulate the release of mediators from the cells for a short period of time and in small quantities. NOS3/cGMP facilitates tumour angiogenesis and signal transduction. Low levels of NO produced by constitutive NOSs prevent leukocyte adhesion, inhibit platelet aggregation, induce immune and inflammatory responses, and regulate cell growth, apoptosis, transduction, and survival. iNOS, expressed in epithelial and immune cells after induction by cytokines or bacterial products, generates large amounts of NO over the long term and cytotoxic products [12,15,28,30]. iNOS mRNA and protein expressed in A498 and A704 RCC cells under hypoxic conditions ensure the hypoxic adaptation of RCC and the ability to form tumour thrombi [31]. NOSi mRNA expressed by mouse RCC macrophages, RENCA, modulates tumour growth and angiogenesis through NO production via microRNA miR-146a [32].

The mechanism of NOS–NO uncoupling is associated with superoxide or hydroperoxide synthesis, leading to reduced NO generation. Disruption of NOS-NO signalling is caused by arginine and BH4 deficiency, NOS dimerisation failure, the presence of methylated and nitrated forms of arginine, glutathionylation, overexpression of ROS and RNS [18].

Post-translational changes mediated by NO (nitrosylation, nitrosation, nitration, transnitrosylation) can give rise to biomolecules with signalling properties. Nitrosylation (direct interaction between NO and transition metals) inhibits mitochondrial cytochrome C oxidase and inactivates Fe-SH and Fe-nonheme sites. Nitrosation (covalent binding of NO(+) to -SH and the formation of S-nitrosothiols) affects redox homeostasis.

Nitration (binding –NO_2_ to organic substrates) modifies the activity of tyrosine in proteins, unsaturated fatty acids, and nucleotides [3,18]. S-nitrosylation plays various regulatory roles in metabolism, apoptosis, protein phosphorylation, and transcription factor activation [30]. S-nitroso groups (SNO) can be generated through transnitrosylation, the transfer of an NO fragment between thiols and S-nitrosothiols (thiol/nitrosothiol exchange) [33]. This mechanism is also the basis for denitrosylation of proteins, which is the necessary process to remove NO from S-nitrosothiols.

Transnitrosylation (transfer of an NO fragment between thiols and S-nitrosothiols) is a process that removes NO from S-nitrosothiols and constitutes a mechanism for denitrosylation of proteins and regeneration of protein thiol groups.

Denitrosylation is catalyzed by S-nitroso glutathione reductase (GSNOR), S-nitroso-CoA reductase (ScoR), and thioredoxin reductase (TrxR). S-nitrosylation is an inhibitory mechanism for crucial enzymes that are involved in cell metabolism [34].

Epigenetic regulation mediated by NO has gained considerable interest in recent years. Under neoplastic conditions, NO influences histone post-translational modifications, DNA methylation, and microRNA status [8,18]. Long non-coding RNAs affect the epigenetic landscape by regulating genes or post-translational modification of proteins, leading to abnormal signal transduction and the induction of malignant transformation in ccRCC [5].

### 2.3. Bimodal Effects of NO in ccRCC

NO levels can serve as an indicator of cellular homeostasis (Figure 1). NO is a primary regulator of tumour progression, with the ability to modulate multiple cellular processes dynamically. Lower concentrations (picomolar to nanomolar range) of NO are present under normal physiological conditions within a cell, but a sudden increase in concentration (micromolar range) results in the development of pathological processes. NO exhibits dichotomous effects on various crucial processes in cancer biology. It is shown to have both pro-tumourigenic and anti-tumourigenic effects, depending on the concentration, source of NO, type, activity, and localization of NOS isoforms, as well as the composition of the tumour microenvironment.

Low NO flux, similar to that generated by constitutive NOSs, maintains cellular homeostasis, drives cell cycle progression, promotes proliferation, neovascularization, angiogenesis, tumour growth and spread, processes governed by the activation of the HIF1α and VEGF signalling pathways [18,30]. Moderate NO levels maintain redox balance in the tumour microenvironment, promote increased invasiveness, metastasis, and cytoprotection, and inhibit apoptosis [18,30].

High NO levels, similar to those generated by iNOS (inducible NOS), induce oxidative and nitrosative stress [18,30]. Elevated NO can lead to DNA damage (single and double-strand breaks, impaired repair), mitochondrial dysfunction, lipid interactions, and the activation of major oncogenic pathways that enhance survival, proliferation, and metastasis in aggressive tumours [18,30].

Additionally, following oxidative reactions, NO can give rise to reactive metabolites, causing the disruption of intracellular and intercellular signalling and the inactivation of transcription factors and effectors within various transduction pathways [5,30].

These bimodal effects of NO emphasize the importance of careful regulation of NO levels, which can be a critical therapeutic target in managing ccRCC.

Similarly, the effects of NO in carcinogenesis are influenced by the metabolic phenotypes of the cells that constitute the tumour microenvironment. The tumour microenvironment consists of cancer cells, stromal cells, immune cells, and endothelial cells, each with distinct metabolic characteristics [9]. NO produced by cancer cells promotes cancer progression, increasing the aggressiveness of these cells in vivo. NOS2 in immune mediators has anti-tumour and pro-inflammatory effects. NO derived from endothelial cells mediates the elimination of disseminated tumour cells, and NO from stromal cells inhibits tumour growth [30].

The subcellular localization and activation of NOSs influence the selectivity of NO targets. NOS1 and NOS3, constitutive isoforms, generate nanomolar concentrations of NO over very short periods, while NOS2, the inducible isoform, produces micromolar concentrations of NO over more extended periods. Most evidence suggests an increase in total NO production in ccRCC patients. This could reflect the activation of iNOS in neoplastic and inflammatory conditions [3,4] The regulation of NOS2 synthesis and production in the tumour microenvironment affects tumour progression.

The level of NO can act as a redox switch. Small amounts of NO regulate anti-inflammatory and antioxidant responses under normal physiological conditions. In contrast, high concentrations of NO in tissues regulate the function of iNOS and promote the oxidation of macromolecules [18].

The NO level is considered to be an indicator of cellular redox status and has an approximate value of 300 nM under physiological conditions [18,30]. Different NOS isoforms in the tumour microenvironment have distinct cellular targets. NO derived from NOSc supports carcinogenesis, while NO derived from NOSi becomes cytotoxic to cancer cells (Figure 1). NO plays a cytostatic/cytotoxic role in cancer cells through multiple mechanisms, including metabolic reprogramming, DNA synthesis inhibition, apoptosis regulation, and necrosis.

NO mediates tumour promotion through genotoxic reactions (DNA damage), anti-apoptotic activity (regulation of caspases and death-associated proteins), regulation of the NO/NOS2–p53 axis, which is critical in defining the apoptotic mechanisms of tumours, upregulation of angiogenesis (production of proangiogenic factors), metastatic actions (overexpression of MMPs and VEGF), and suppression of the immune response (reduction in leukocyte infiltration) [30,35].

NOSs–NO plays a significant role in tumour suppression [9,35]. iNOS is essential for the development, maturation, and differentiation of T cells, B cells, monocytes, and dendritic cells [35]. In response to tumour progression, NO and RNS stimulate the passage of immune cells from the bloodstream around cancer cells, the polarization of tumour-associated macrophages (TAMs), the transactivation of eNOS/iNOS, the stabilization of HIF1α, the transcription of proangiogenic factors, the activation of S-nitrosylation, the inactivation of Janus kinase 3, early response kinase, and protein kinase B, which prevent the IL-2 response.

Immune-activating M1 macrophages and immune-suppressing M2 macrophages differentiate based on the presence of arginase and iNOS. Both arginase and iNOS catalyze arginine in different ways (Figure 1. M1 macrophages, with high iNOS expression, produce large amounts of NO and use NO/RNS for cytotoxic clearance. The host’s NO/NOSi-dependent defence mechanism associated with M1 macrophages provides survival benefits to cancer cells and supports malignancy [9,35].

NO adapts the metabolism to the needs of tumour cells by inhibiting metabolic enzymes and regulating mitochondrial modulators involved in cancer-associated growth, antioxidant responses, and metabolic rewiring. NO regulates the electron transport chain complexes (cytochrome c oxidase, complex IV), enzymes in the tricarboxylic acid cycle (aconitase, α-ketoglutarate dehydrogenase, succinate dehydrogenase), enzymes involved in fatty acid oxidation, branched-chain amino acid metabolism, pyruvate kinase M2, and the stability of the TRAP1/SIRT3 complex (Tumour Necrosis Factor Receptor-Associated Protein 1/sirtuin 3). At low concentrations, NO competes for the catalytic site, whereas at high concentrations, inhibition occurs through oxidative post-translational modifications. NO generated by NOS1 activates SIRT3, while NO generated by NOSi inactivates SIRT3. These mitochondrial events simultaneously provide the capacity to cope with oxidative stress and adapt to metabolic changes [34,35,36,37,38,39].

### 2.4. Quantitative Determination of NO Metabolites in Biological Samples

The quantitative determination of NO in vitro and in vivo is particularly challenging due to its rapid diffusion through membranes, biological interferences, a pronounced tendency to auto oxidize, short lifespan (seconds), low equilibrium concentration (nanomoles), and the heightened reactivity of NO and its natural derivatives (NO, NO_2_, N_2_O_3_, NO_2_^-^, which rapidly react with haemoglobin, glutathione, sulfhydryls, and unsaturated fatty acids).

Various techniques have been developed for measuring NO and its metabolites in biological systems. The primary methods for quantifying NO and its derivatives include [40,41]:

1. Spectrophotometry: Utilizing azoic dyes and the Griess test.
2. Fluorescence: Employing reagents such as Diaminofluorescein (DAF-2).
3. Luminescence: Using luciferin–luciferase assays.
4. Electrochemical: Employing amperometric NO microelectrodes.
5. Tandem Mass Spectrometry: Including mass spectrometry in tandem (MS/MS) and electrospray ionization mass spectrometry (ESI-MS/MS).
6. Liquid Chromatography-Mass Spectrometry (LC-MS): Combining liquid chromatography with mass spectrometry
7. Electron Paramagnetic Resonance (EPR): Employed for NO detection.
8. HPLC: High-Performance Liquid Chromatography is another technique.
9. Antibody-Based Methods: These encompass immunohistochemical, immunoblotting, and enzyme-linked immunosorbent assays (ELISAs).
10. Chemiluminescence: A method based on the detection of light emission.
11. UV-Visible Absorption Spectrum: Measuring the absorption of UV-visible light.

Accurate identification and quantification of the rate of NO formation or degradation, precursors, derived species, molecular targets, and the consequences of disturbances in the NO cycle and related molecular pathways, alongside pharmacological and omics methods, will help solve the role of NO in ccRCC (Table 1).

We observed systemic alterations in the levels of NO metabolites (NO_3_^−^, NO_2_^−^, NO_x_ = NO_3_^−^ + NO_2_^−^) and overproduction of nitrotyrosine in cancer patients. The aberrant activation of NO signalling induced by the hypoxic microenvironment is associated with tumour progression [3,4].

The profile of NO (NO) in tumour cells and the microenvironment influences the rate of cancer progression, therapy effectiveness, and patient prognosis. In a recent study, it was noted that markers of nitrosative stress (3-nitrotyrosine, nitrite/nitrate) were elevated in the ccRCC group, correlating with the increased pathological stage of the tumour (TNM, histological grade, angioinvasion) [74].

## 3. Dysregulated Ureagenic Cycle—A Distinctive Sign in ccRCC

**Figure 2:**
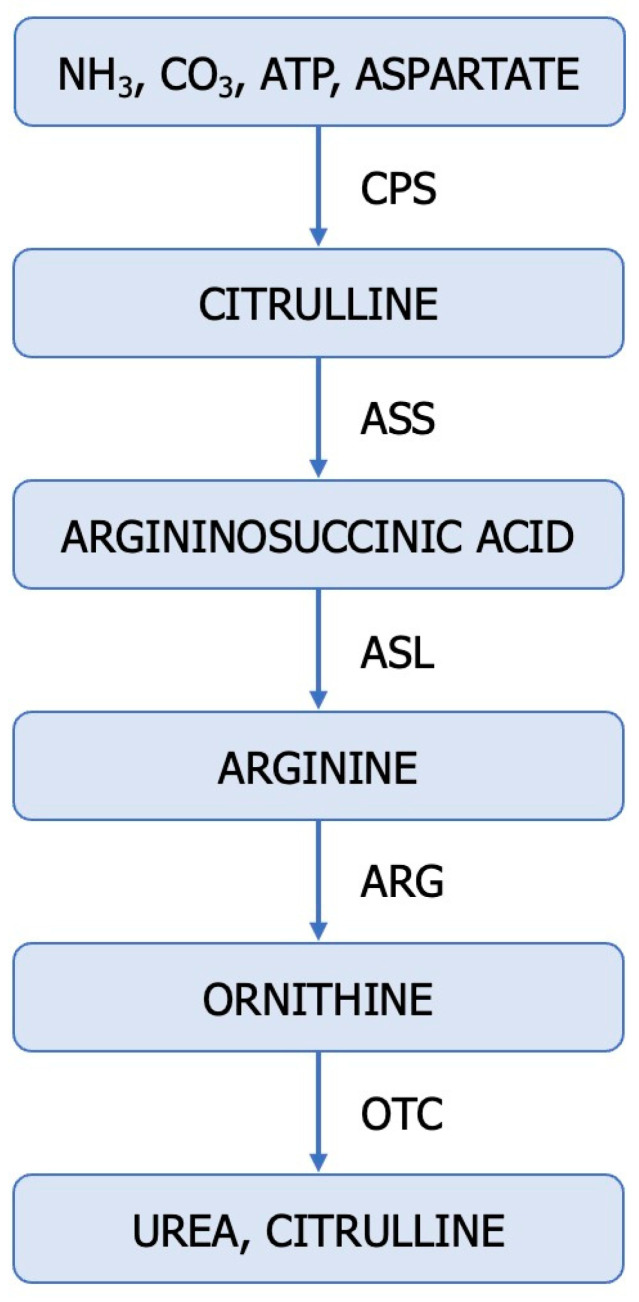
The ureagenic cycle: CPS-1—carbamoyl-phosphate synthetase I (E.C. 6.3.4.16); OTC, ornithine transcarbamylase (E.C. 2.1.3.3); ASS—argininosuccinate synthetase (E.C. 6.3.4.5); ASL—argininosuccinate lyase (E.C. 4.3.2.1); ARG—arginase (E.C. 3.5.3.1).

The fact that the dysregulation of the ureagenic cycle represents a distinctive feature of ccRCC is supported by the low levels of mRNA and the altered expression and function of representative enzymes (ASS1, ASL, ARG2) in tumour samples compared to normal kidney tissue [46]. The urea cycle is a metabolic pathway responsible for converting excess nitrogen from ammonia and aspartate into urea (Figure 2).

Disruptions in the urea cycle arise from enzymatic deficiencies and can additionally be induced by the dysfunction of transporters responsible for transferring mitochondrial aspartate into the cytosol. The aforementioned changes in enzyme expression and metabolites within the urea cycle at different stages of cancer development demonstrate the dynamic alterations in this metabolic cycle [75,76,77,78].

Some enzymes in the urea cycle are repressed in primary renal cancer cells, while ASS1 is epigenetically reactivated in metastatic populations [1]. Sensitive to the arginine levels in the microenvironment, the selective expression of ASS1 provides metastatic renal cancer cells with the ability to utilize nitrogen from BCAA catabolism to produce arginine [1]. Reduced ARG2 activity promotes ccRCC tumour growth through at least two distinct mechanisms: preserving pyridoxal phosphate and preventing the accumulation of toxic polyamines [7].

In conclusion, the dysregulation of the urea cycle in cancer is accompanied by the inactivation of ASS1, activation of CAD (carbamoyl-phosphate synthetase 2, aspartate transcarbamylase, and dihydroorotase), overexpression of NO, enhanced pyrimidine synthesis at the expense of purine, mutagenesis, increased cell proliferation, invasiveness, and survival, poor prognosis, and increased immunotherapy efficacy [1,10,76,78]. Disruption of urea cycle enzymes in ccRCC contributes to the rerouting of carbon and nitrogen towards the generation of anabolic biomass [46]. Dysregulation of the urea cycle is a metabolically advantageous phenomenon for cancer cell proliferation.

## 4. The Upregulation of Glutamine: An Alternative Source of Nitrogen for ccRCC

**Figure 3:**
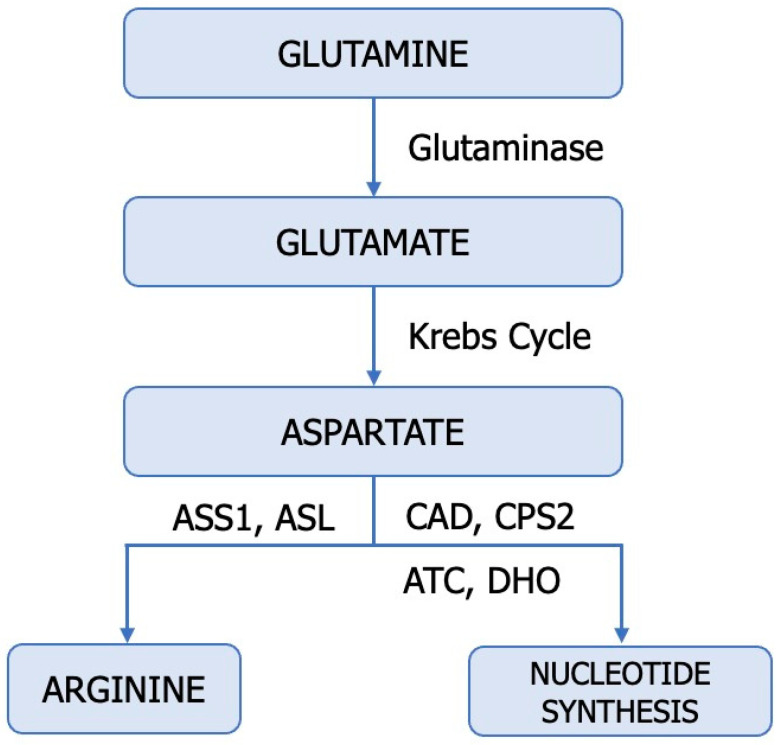
Utilization of glutamine in tumours. ASL—argininosuccinate lyase (E.C. 4.3.2.1); ASS1—argininosuccinate synthase (E.C. 6.3.4.5), CAD—trifunctional protein, CPS 2—carbamoyl-phosphate synthase 2 (E.C. 6.3.5.5), ATC—aspartate transcarbamylase (E.C. 2.1.3.2) DHO—dihydroorotase (E.C. 3.5.2.3).

Amino acids such as glutamine, arginine, aspartate, alanine, glycine, and serine serve as significant nitrogen sources for cancer cells, immune cells, endothelial cells, and stromal cells within the tumour microenvironment (Figure 3) [9]. Cancer cells metabolize glutamine (Gln) differently than normal cells, requiring an enzyme known as glutaminase (E.C. 3.5.1.2). ccRCC cells exhibit higher levels of glutamine, glutamate, and the SLC1A transporter compared to normal renal tissue [79]. Hypoxic cells, particularly those with a VHL deficiency, employ glutaminase to obtain glutamate, which indirectly plays a pivotal role in pyrimidine and lipid synthesis as well as antioxidative processes (Figure 2). Human ccRCC renal cells utilize HIF2α-mediated reductive carboxylation to sustain de novo pyrimidine biosynthesis [80,81,82]. Recent studies suggest a reciprocal regulation between Gln and NO [83]. Gln, an essential amino acid, competitively inhibits Cit availability for interacting with L-Arg, thereby regulating macrophage NO production capacity.

Moreover, Gln modulates NO synthase to catalyze the conversion of Arg to NO. Glutamine appears to be a significant modulator interfering with citrulline-mediated NO production [80,81,84]. On the other hand, activated glutamate receptors and NO reduce glutamine synthetase (E.C. 6.3.1.2) activity, while Gln inhibits ASS activity and NO synthesis. Through this mechanism, glutamine decreases intracellular arginine concentration and NO release. The importance of citrulline and glutamine concentrations in NO synthesis must be interpreted with caution, as both metabolites can be modulated through independent NOS mechanisms [83]. As a newly discovered molecule, mitochondrial protein Sirtuin 4 (SIRT4) has been linked to alternative glutamine metabolism and regulation of the tumour microenvironment. In ccRCC cells, SIRT4 promotes apoptosis by increasing ROS. Downregulation of SIRT4 in ccRCC promoted HO-1 expression in hypoxic cells, counteracting the pro-apoptotic effect of SIRT4. Moreover, SIRT4 regulates ROS and HO-1 expression through Akt and P38MAPK phosphorylation in ccRCC [5].

The nucleotide imbalance in tumours is associated with multiple transversions that propagate from DNA to RNA and proteins, leading to the production of immunogenic neoantigens [10]. Pyrimidine synthesis plays a significant role in carcinogenesis, affecting the prognosis of patients and their response to immunotherapy.

## 5. Cellular Arginine Depletion—A Proliferation Strategy in ccRCC

**Figure 4:**
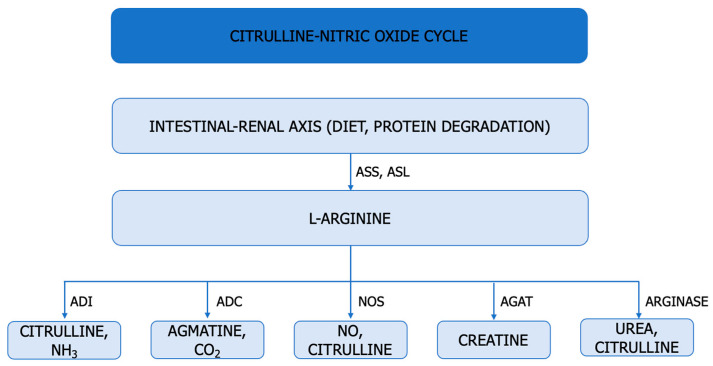
Arginine Metabolism. ASS (E.C. 6.3.4.5: argininosuccinate synthetase, ASL (E.C. 4.3.2.1): argininosuccinate lyase, ADI (E.C. 3.5.3.6.): arginine deiminase, ADC (E.C. 4.1.1.19) arginine decarboxylase, NOS (E.C. 1.14.13.39): NO synthase, AGAT (E.C. 2.1.4.1.) arginine:glycine amidinotransferase.

A significant metabolic defect in tumour biology is represented by the altered intrinsic ability of cancer cells to synthesize arginine. In tumour metabolism, arginine plays a role in signalling, epigenetic regulation, and immunomodulation [14,85]. Arginine is a conditionally essential amino acid synthesized under conditions of rapid cell growth through two pathways: the citrulline–NO cycle and the intestinal–renal axis, utilizing ASS and ASL. Arginine is metabolized into NO and citrulline by NOS, into agmatine and CO_2_ by ADC, into ornithine and urea by arginase, into creatine by AGAT, and citrulline and NH_3_ by ADI (Figure 4) [22].

Arginase 1 is preferentially expressed in the liver, while arginase 2 is primarily identified in the kidney. In renal carcinogenesis, arginine supports rapid tumour growth and induces T-cell dysfunction [66,75]. Arginine regulates NO production in cancer cells [21].

Arginine regulates NO production in cancer cells [21]. In renal carcinogenesis, arginine facilitates rapid tumour growth and induces T-cell dysfunction [66,75].

ASS and ASL, which are cytosolic enzymes involved in nitrogen metabolism, impact ccRCC growth by depleting cellular aspartate reserves and disrupting pyrimidine production. Loss of ASS1 promotes cancer proliferation by diverting its substrate, aspartate, toward pyrimidine synthesis (carbamoyl-phosphate synthetase 2 (CPS2), aspartate transcarbamylase (ATC), and dihydroorotase [10]. Metastatic renal cancer cells reactivate ASS1, an enzyme suppressed in primary ccRCC, in order to maintain the invasive potential of metastatic renal cancer cells in vitro and in vivo and to modulate the sensitivity of metastatic cancer cells to arginine depletion [1].

Arginase 2, preferentially expressed in the kidneys, plays a significant role in the proliferation of tumour cells and the reduction in L-arginine availability [66,67]. Reduced ARG2 activity has been found to promote ccRCC tumour growth through multiple mechanisms. Arginase 2 suppresses renal carcinoma progression by depleting the biosynthetic cofactor pyridoxal phosphate and increasing polyamine toxicity.^7^ Cancer cells rely on glutamine for survival and proliferation. VHL loss-dependent reprogramming of Arg is necessary to maintain the NO reservoir in cancer cells [7].

## 6. Hyperammonemia in ccRCC

The catabolism of glutamine and other amino acids is accompanied by the secretion of ammonia, leading to the loss of amino groups from the cell and the accumulation of ammonia in the tumour microenvironment [47,48,78,86]. Extracellular ammonia is viewed as either a toxic cellular by-product of amino acid metabolism that needs to be metabolized into a non-toxic form, such as urea, for excretion from the body or recycled into the central amino acid metabolism to maximize nitrogen utilization and support tumour biomass [47,78,87].

The accumulation of ammonia has allowed glutamate dehydrogenase to function in reductive amination, accelerating the incorporation of nitrogen from ammonia back into amino acids (Figure 4).

The progression of ccRCC depends on changes in ammonia metabolism [7,48]. Ammonia accumulates in the tumour microenvironment and induces metabolic reprogramming of T cells. Increasing ammonia clearance reduces tumour size, enhances survival, reactivates T cells, and improves the effectiveness of anti-PD-L1 therapy [7,48]. Recently, it has been reported that during axitinib treatment for metastatic renal carcinoma, hyperammonemia developed due to associated thyroid disorders [88].

Ammonia activates NO production by stimulating arginine uptake and regulating the distribution of ADMA and SDMA. The intracellular L-Arg/ADMA ratio modulates iNOS activity and NO levels [89]. These findings warrant further investigations into the role of ammonia as a modulator of NO production.

## 7. The Reduction in BCAA Catabolism in ccRCC

**Figure 5:**
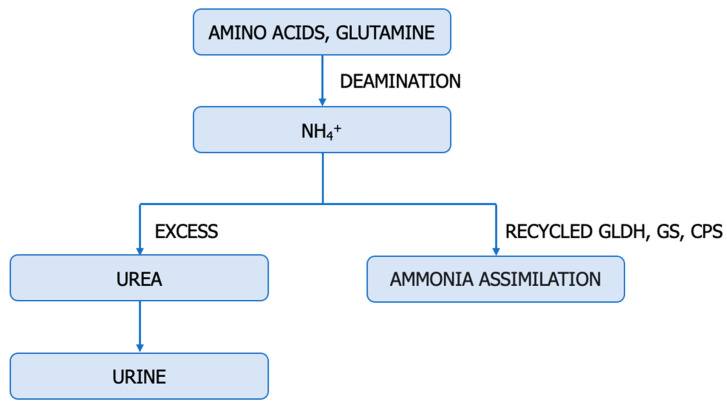
Ammonia metabolism. GLDH—Glutamate dehydrogenase (E.C. 1.4.1.3), GS—Glutamine synthetase (E.C. 6.3.1.2), CPS—Carbamoyl phosphate synthetase (E.C. 6.3.4.16).

**Figure 6:**
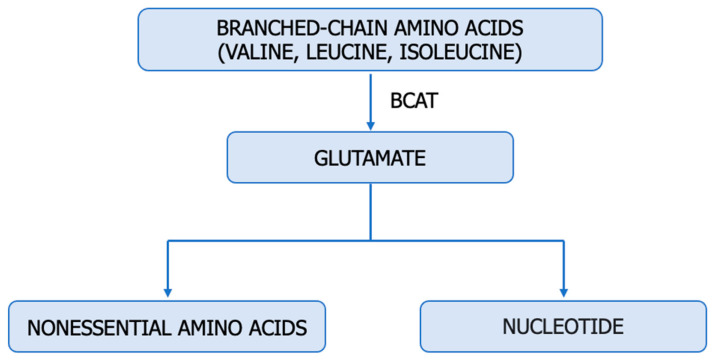
BCAA metabolism in cancer. BCATs—branched-chain amino acid transaminases (E.C. 2.6.1.42).

Branched-chain amino acids (BCAA), namely valine, leucine, and isoleucine, belong to a group of essential amino acids. BCAAs can be directly incorporated into proteins or participate in other essential metabolic pathways for tumourigenesis [14,90]. In VHL-deficient renal cancer cells, BCAA catabolism represents an indirect source of nitrogen for nucleotide and non-essential amino acid biosynthesis (Figure 5) at all stages of tumour evolution [1,14]. BCAA catabolism in renal cancer cells is linked to the glutamate–glutamine axis, transcriptional sensitivity to VHL restoration, epigenetic modification, enhanced tumour immunity, and ferroptosis [14]. Hypoxia suppresses BCAA catabolism in specific tissues but regulates the expression of SLC7A5 and BCAT1 in tumours [40,62]. BCAT1 and BCAT2, which are responsible for BCAA degradation, can be considered immunosuppressive factors affecting the cancer cell’s survival ability via the NO cycle (Figure 6) [91].

The decrease in glutamate derived from BCAAs can be compensated by increasing glutaminase activity, which generates glutamate from glutamine. The inhibitory effect of BCAAs on lipid peroxidation and NO scavenging activity allows the development of antioxidant and anti-inflammatory products in the pharmaceutical industry [92].

## 8. Endogenous Inhibitors of NO Synthesis

Methylarginines and DDAH enzymes are endogenous modulators of NO production. Methylarginines are endogenous metabolites obtained through the post-translational N-methylation of arginine residues incorporated into proteins, catalyzed by protein methyltransferases (PRMT), and released into the cytosol following proteolysis. Cationic amino acid transporters (CAT1, CAT2A, and CAT2B) facilitate the transport of methylarginines across cell membranes. The primary methylarginines include monomethylarginine (NMMA), asymmetric dimethylarginine (ADMA), and symmetric dimethylarginine (SDMA). ADMA and NMMA compete with L-arginine for binding to the active site of NOS, acting as competitive inhibitors for all isoforms. SDMA competes with CAT transporters for L-arginine, indirectly exerting inhibitory effects on NO synthesis [3,4,24,26,89].

**Figure 7:**
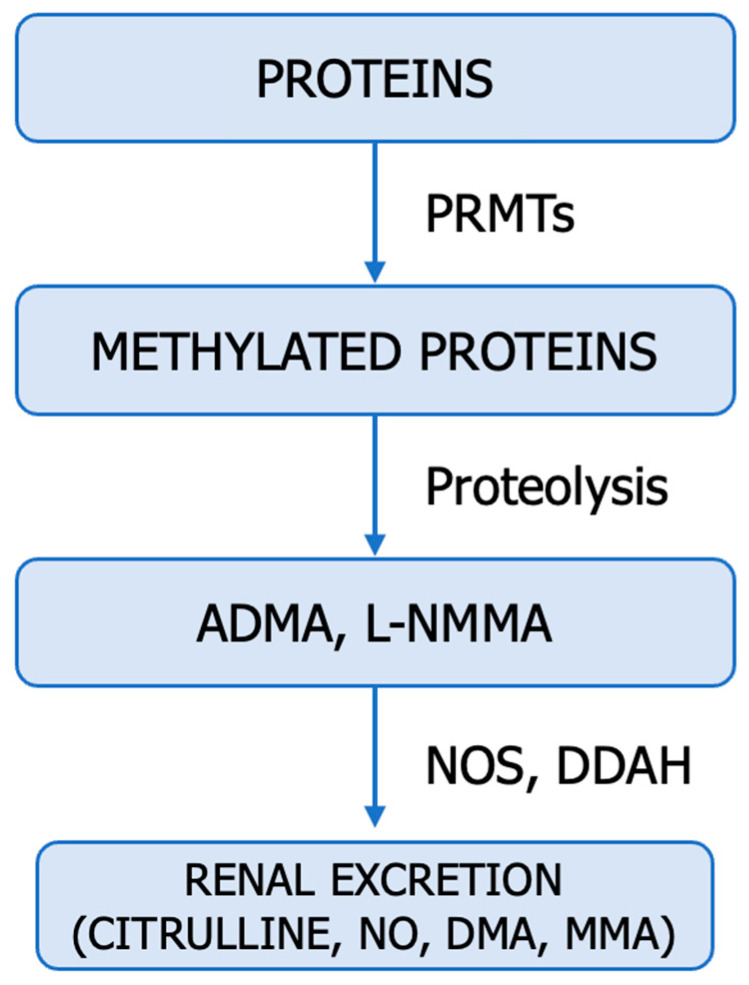
PRMT–DDAH–ADMA–NO Axis. PRMT1 (E.C. 2.1.1.319)—protein–arginine–methyltransferase, DDAH (E.C. 3.5.3.18)—dimethylarginine dimethylaminohydrolase, NOS (E.C. 1.14.1,39.)—NO synthase, ADMA—asymmetric dimethylarginine, NMMA—monomethylarginine, DMA—dimethylamine.

Enzymes dimethylarginine dimethylaminohydrolases (DDAHs) metabolize ADMA and NMMA into L-citrulline, dimethylamine, or monomethylamine. They are vital components in maintaining the homeostatic control of NO [12,26,93]. The DDAH/ADMA/NO pathway is summarized in Figure 7.

The pathophysiology of increased ADMA and SDMA in malignancies can have a complex origin. The rate of synthesis and degradation, inflammation, oxidative stress, antioxidants, redox status, proteinuria, endothelial function, functional activity of PRMTs, and DDAHs influence the circulating levels of methylarginine [3,4,24,89].

The dysregulation of the DDAH/ADMA/NO pathway, resulting in locally increased NO availability, is often associated with promoting tumour angiogenesis, growth, invasion, and metastasis. These findings warrant further investigation into the role of the PRMT–DDAH–ADMA axis in NO production [12,89].

## 9. The Inactivation of VHL and the Accumulation of HIFs—Essential Characteristics of ccRCC

**Figure 8:**
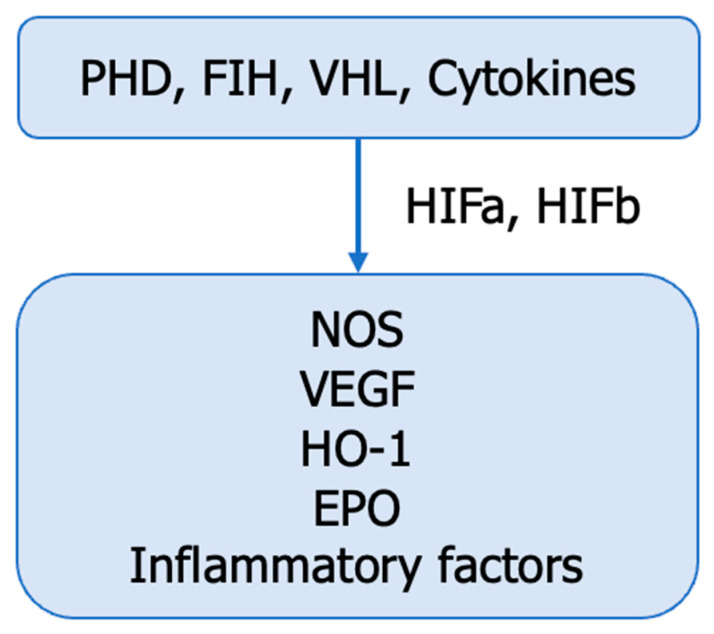
VHL—HIFs–NOS interaction. HIF—hypoxia-inducible factors; PHD—prolyl-4-hydroxylases (E.C. 1.14.11.2); FIH—factor–inhibiting HIF, VHL—von Hippel—Lindau protein, EPO—erythropoietin, VEGF—vascular endothelial growth factor, HO-1—heme oxygenase-1 (E.C. 1.14.14.18), NOS—NO synthase (E.C. 1.14.1,39), GLUTs—glucose transporters.

ccRCC, whether sporadic or genetic, is characterized by frequent mutations in genes located on chromosome 3p, including VHL, PBRM1, BAP1, SETD2 H3K36, KDM6A, KDM5C, PTEN, mTOR, PIK3CA, TP53 [6]. The inactivation of VHL is accompanied by the accumulation of hypoxia-inducible factors (HIFs) in the tumour microenvironment, their interaction with NO synthases, the regulation of intratumoural NO production, and the development of renal tumours (Figure 8) [94].

HIFs (HIF1, HIF2, HIF3) are heterodimers composed of alpha and beta subunits with distinct spatial distributions, specific inductions, and well-defined biological roles during tumour hypoxia (Figure 8). HIF-1α is primarily located in the renal tubular epithelium, while HIF-2α is mainly found in stromal and endothelial cells. HIFs play a dual role in the hypoxic microenvironment of tumour cells. HIF-1α is involved in the acute phase, while HIF-2α and HIF-3α are more frequently involved in chronic hypoxia. HIF-2α is considered an oncogene in ccRCC, whereas HIF1α likely has a tumour-suppressive function. However, a finer balance between HIF1α and HIF2α has also been proposed. HIF1α is essential in tumour initiation and glucose metabolism reprogramming, while HIF2α regulates biosynthetic pathways such as lipid metabolism, ribosome biogenesis, and the transcriptional activity of numerous factors. HIF-α combines with HIF-β in the nucleus to mediate the transcription of target genes. Moreover, both HIFs are crucial for the immune reprogramming of ccRCC tumours [6,94].

Many studies have suggested that HIF-1α can inhibit tumour growth while HIF-2α promotes tumour growth and metastasis [94]. HIF activation suppresses ASS1 and redirects aspartate toward nucleotide biosynthesis. In the metastatic population [1]. NOSs (endothelial, neuronal, inducible), upregulated by HIFs, promote NO release into the tumour microenvironment.

## 10. NO-Based Therapy for ccRCC

The interactions between cancer cells and the tumour microenvironment play crucial roles in the progression of ccRCC. By producing growth factors or cytokines and accentuating hypoxia and necrosis, RCC cells can promote the attraction and activation of non-tumour cells. Recent studies have identified a subpopulation of CD133+/CD24+ cells in ccRCC specimens that exhibited self-renewal capacity and clonogenic multipotency. These cells are referred to as “cancer stem cells” (CSC), utilizing signalling pathways similar to those that control cell fate during early embryogenesis [95,96]. As discussed in this article, the NO/NOS system is interconnected with various tumour-promoting processes in the tumour microenvironment. In recent years, numerous studies have reported the design and development of NO-based nanomedicines, with the intention of being used in cancer treatment. In this regard, current and future objectives of pharmacotherapy are focused on the development of NO donors encapsulated in liposomes, antibody-NO conjugates, NO donors encapsulated in exosomes, iNOS overexpression (gene therapy), and tissue-selective donors that release NO in a controlled manner. Delivered at high concentrations, NO inhibits tumour growth and enhances apoptosis/ferroptosis of tumour cells by generating both reactive oxygen species and reactive nitrogen species [97,98].

## 11. Conclusions and Future Perspectives

In recent years, the study of the tumour microenvironment has gained significant attention [2,4,13]. Studies on tumour microenvironment metabolism have revealed that altering cells’ metabolic profiles provides a survival advantage for growth and survival under challenging conditions.

Excessive NO production is primarily attributed to inducible NO synthase (iNOS) induction during oxidative stress and inflammatory states, which play a crucial role in ccRCC development. This article observed that the reprogramming of NO metabolism and the failure of immune surveillance in preventing malignant conditions are crucial factors in ccRCC progression.

Patients with ccRCC develop disturbances in most of the NO synthesis metabolites, worsening with ccRCC progression. NO metabolites may serve as promising markers for ccRCC stage and severity. Further research is needed to establish their clinical potential. NO affects both tumour and immune metabolism and exerts a significant regulatory effect on progression and immunotherapy. Therefore, the study aims to determine the metabolic characteristics of NO metabolites, which could help identify molecular features that provide opportunities for targeted metabolism. Clarifying these issues will contribute to developing personalized medicine for ccRCC patients. In recent years, numerous studies have reported the design and development of NO-based nanomedicines. These include NO donors encapsulated in liposomes, antibody–NO conjugates, NO donors encapsulated in exosomes, ligands selective for NOS isoforms, and tissue-selective donors that release NO in a controlled manner.

## Figures and Tables

**Table 1:** No Synthesis Metabolites in ccRCC.

NO Parameters (References)	Biological Systems	Results	Conclusions
Calcium-dependent and calcium-independent NO synthetase [42]	Human kidney/RCC, proximal tubular cell lines HN4, HN51.	Calcium-dependent NOS activity, identified in all the samples studied, was downregulated in RCC compared to non-malignant renal tissues studied; calcium-independent NOS activity was inconsistently expressed in the renal tissue.	NO exerted cytostatic effects on cultured renal cells.
NOS1, NOS2, NOS3 [43]	Non-neoplastic renal tissues and RCC	In non-neoplastic tissues, NOS3 immunoreactivity was increased and NOS2 was reduced compared to RCC. The NOS expression was correlated with tumour size and a poor prognosis.	NOS3 as a predictive factor in RCC
NOS, sGC, nitrotirosine [44]	Normal and tumoural renal tissue (benign and malignant tumours).	NOS1 is downregulated in malignant tissues and associated with the tumour grade; sGC is present in all renal tumours; nitrotyrosine is present in normal renal parenchyma and tumour tissues.	Autocrine signalling of NO is similar in normal and non-malignant renal tissues and altered in malignant tissues
Nitrites [45]	Serum (apparently healthy women diagnosed with RCC)	Elevated serum level in patients with RCC	Elevated serum nitrite levels are associated with a low risk of renal cancer.
ASS1, ASL, Arg2 [46,47]	RCC tissue samples and control	mRNA and ASS1, ASL, Arg2 activity are reduced in RCC vs. normal kidney Altered urea cycle-metabolic pathway in RCC.	Attenuation of the cytotoxic effects of NO. ASS1, ASL, Arg2 -metabolic suppressors in RCC.
NOSi-ARN [31]	RCC and control tissue samples	mRNA and iNOS protein present in tumour thrombi in patients with RCC and in A498 and A704 cells under hypoxic conditions.	It mediates the formation of tumour thrombi and hypoxic adaptation.
Arg2, ASS1 [46,48]	Normal and malignant renal cell lines	The expression of enzymes in the urea cycle is downregulated in RCC compared to the control. Deficiency of enzymes in the urea cycle disrupts polyamine synthesis, conservation of pyridoxal phosphate, arginine auxotrophy, infiltration of cytotoxic T cells in the tumour tissue, and immunosuppression in the tumour microenvironment.	Arg2 and ASS1 are potential metabolic suppressors of renal tumourigenesis.
ASS1, ADI (E.C.3.5.3.6) [35]	Biopsy samples, animal models, cell lines.	Low or undetectable ASS1 in RCC, present in normal proximal tubule epithelium. Exogenous ADI (arginine deiminase) determines antiproliferative and antiangiogenic effects in vivo on RENCA tumour cells and extends the survival of tumour-bearing mice.	Arginine deprivation via ADI—an antitumour strategy in RCC
Spermine, spermidine [49]	Normal and malignant human renal tissue	Spermidine levels and spermidine/spermine ratio increase; normal tissue < differentiated RCC < poorly differentiated RCC. The other polyamines do not show differences between normal tissues, tumours, and metastases.	Polyamines—biochemical markers for the malignancy of RCC
Diamine, spermidine, spermine [50]	Tissue, urine, blood	Elevated levels are correlated with the progression of RCC	Polyamines—tumour markers in RCC
Agmatinase (E. C.) [51]	Normal and malignant renal tissue	The expression and mRNA of agmatinase are decreased in RCC compared to benign renal tumours. Accumulated agmatine stimulates NOS3 and NOS2, leading to NO synthesis.	Reduced agmatinase increases the cytotoxic activity of NO in RCC
RNS, NO_2_^−^ [52]	Cell cultures	JS-K, a NO donor, stimulates the increase in ROS (Reactive Oxygen Species) and RNS (Reactive Nitrogen Species), the reduction in GSH/GSSG (glutathione redox status), the increase in pro-apoptotic proteins (Bak, Bax), and the reduction in anti-apoptotic proteins (Bcl-2) in RCC (Renal Cell Carcinoma). JS-K induces apoptosis in cancer cells by modulating the production and signalling of NO, MAPK (Mitogen-Activated Protein Kinase), the ubiquitin–proteasome pathway, and the β-catenin/T-cell factor (TCF) signalling pathway	NO released by JS-K induces apoptosis in renal carcinoma cells by increasing the levels of ROS/RNS and induces chemosensitivity of tumour cells to doxorubicin
NOS, cGMP [53]	Experimental and human tumours.	The NOS enzymes are overexpressed in tumour tissues.	NO metabolites correlate with angiogenesis and tumour aggressiveness.
Dietary nitrite, nitrate intake [54]	Evaluation of nitrates and nitrites in food sources (41 articles, 13 types of cancer).	Nitrates from plant sources and nitrates in general do not affect the development of renal cancer. Nitrites from processed meat are associated with an increased risk of renal cancer < pancreatic cancer < thyroid cancer, stomach cancer, glioma, and others.	The nitrites/nitrates intake has specific effects on the type and site-specific risk of cancer
Phytonutrients [55,56,57,58,59,60]	Epidemiological studies on the role of dietary factors.	Phytonutrients play an essential role in cancer prevention. Dietary sources of nitrites and nitrates have a role in immunity and vascular function. Dietary sources of nitrites include vegetables, fruits, and processed meat.	Vitamin C inhibits endogenous nitrosation.
Red and processed meat [61]	Meta-analysis (12 case-control studies, 16 cohorts)	No statistically significant data were obtained between the consumption of red and processed meat, individual variables (BMI, smoking, total energy intake), and the development of renal cancer.	An independent relationship between meat consumption and the risk of renal cancer was not evident.
Red and processed meat [62]	Meta−analysis (23 eligible publications) on the association and impact of red meat consumption on RCC	Positive relationship between consumption of beef, salami, ham, bacon, sausages, hamburgers, and renal cancer.	Statistically significant positive association between red meat consumption and RCC
Dietary factors [63,64]	Report on 22 meta−analyses (566 publications).	No suggestive or convincing evidence between the consumption of foods, beverages, alcohol, macronutrients, micronutrients, and the incidence of RCC	The intake of vegetables and vitamin C is associated with the risk of RCC
Serum NO_2_^−,^ NO_3_^−^ [65]	RCC patients and control patients	No significant differences between patients and controls. Variations depending on the tumour grade.	NO exerts immunoregulatory effects in RCC
Arginase 2 [66]	Murin renal cell lines, normal and neoplastic.	Arginase 2 rapidly metabolizes L-arginine, suppresses tumour growth, and reduces the expression of CD3zeta.	Arginase 2 modulates the function of T cells, depleting arginine.
Arginase 2 [67]	Peripheral blood of metastatic RCC and control patients	Myeloid suppressor cells producing arginase present in patients with metastatic ccRCC	Arginase 2 regulates the availability of arginine.
Arginase 2 [7]			Arginase 2 supports the growth of ccRCC
BCAA, BCAT, ASS1 [1,21,68,69,70]	Cultured primary and metastatic renal cancer cells (omic study)	Transcriptionally suppressed BCAA catabolism, overexpressed BCAT, urea cycle, glutathione, cysteine/methionine, arginine, glutamine, tryptophan, reactivated polyamines in ccRCC show metabolic flexibility during tumour progression and offer invasive potential.	Altered metabolic advantage for cancer cell survival
SIRTs [34,36,71,72,73]	ccRCC cells, cell lines, genetic models, pharmacological models, omic studies, computational	SIRTs-1,3,6,7 maintain renal homeostasis. SIRT1 regulates NOSe in glomerular cells. NO regulates the stability of the SIRT3/TRAP1 complex. SIRT3 has dual effects on tumour growth. SIRT3 has antioxidant and anti-inflammatory effects in kidney disease. SIRT4 inhibits glutamine metabolism	Activating SIRTs before tumour initiation is a preventive strategy. NO/SIRT3 regulates mitochondrial biogenesis in ccRCC and cell sensitivity to antitumour therapy.
